# Personal pathways to success: an innovative program to overcome cancer patient barriers to tobacco cessation and promote patient participation

**DOI:** 10.1007/s00520-026-10971-w

**Published:** 2026-07-03

**Authors:** Cary A. Presant, Brenda Gascon, Sophia Yeung, Jonjon Macalintal, Argelia Sandoval, Dan Raz, Loretta Erhunmwunsee, Janet Cronkhite, Khristie Davy, Mary Cianfrocca, Ravi Salgia, Yuman Fong, Kimlin Tam Ashing

**Affiliations:** City of Hope National Medical Center, 1500 E. Duarte Rd., Duarte, CA 91010 USA

**Keywords:** Smoking cessation, Barriers to tobacco cessation, Tobacco cessation services, Patients from minority groups, Personalizing tobacco cessation

## Abstract

**Purpose:**

Tobacco cessation is an important supportive component of cancer care. We developed a novel program to overcome cancer patient barriers to tobacco cessation and improve outcomes.

**Methods:**

The personal pathways to success (PPS) program consisted of core components of a motivational interview, an instructional video on tobacco cessation, referral to cessation consultation, and/or an offer of cessation medications. Patients who agreed to participate in PPS were also offered the choice of 30 additional PPS supplemental support cessation services. Participants were active smokers before planned surgery at City of Hope (COH) and were referred to the PPS program. We compared tobacco use in patients who participated in PPS with supplemental support services to patients who did not participate. We compared non-Hispanic white patients to patients from minority groups. Evaluations of tobacco use were performed before surgery in all patients, and at 30 days, 3, 6, 9, and 12 months after surgery in PPS participants, and at 12 months in non-participants.

**Results:**

Among 70 patients, 7 were re-directed by insurance to a non-COH provider. Among the other 63 patients, 58 completed the motivational interview (acceptability rate 92%). Thirty-five of 63 patients (56%) agreed to participate in PPS with supplemental support services. There was equal use of services in non-Hispanic white patients and in patients from minoritized backgrounds. Abstinence preoperatively was 17% in participants and 6% in non-participants. At 12 months after surgery, 42% of participants were abstinent versus 17% of non-participants (*p* = 0.11). At 12 months after surgery, 55% of participants were abstinent from combustible tobacco use (abstinent or only vaping) compared to 22% of non-participants (*p* = 0.037).

**Conclusions:**

The PPS program is feasible. Participation is associated with reduced cigarette use among cancer patients. Further studies are warranted.

**Implications:**

Allowing cancer patients to choose among a defined menu of supplemental cessation services offers a method to support patients in their goal of quitting.

## Background

Tobacco use is a major health hazard in the general population and results in disabilities, diseases, cancers, and shortened length of life [1]. Effective tobacco cessation treatment is an important component of cancer care and has been described as the Fourth Pillar of Cancer Care [2]. In oncology patients, tobacco use is associated with development of second cancers, lower chance of cure, increased morbidity of treatment, reduced benefit of treatment, and worse outcomes [3].

At City of Hope (COH), we have implemented a comprehensive tobacco cessation strategy [3]. Our previous multidisciplinary program was built on identification of active smokers by using a tobacco use survey (TUS), evaluation by oncologists and hematologists for referral to the cessation clinic, initial telephone contact and registration by a tobacco treatment specialist, scheduling a cessation counseling session, and prescribing cessation medications. In addition, we have introduced innovations in tobacco cessation efforts including outreach initiatives to underserved communities [4], development of a collaborative network with loco-regional agencies [4], and naming medical and nursing tobacco cessation champions for clinics and community cancer care offices [5].

There are many patient-specific barriers to tobacco cessation [4, 6]. These include lack of education/knowledge about smoking risks and benefits of cessation, lack of access to cessation services, lack of insurance coverage, stress, anxiety, high cost of medications, and cultural and/or language obstacles.

Large literature reviews have studied barriers to tobacco cessation in healthcare or community settings but not specific to cancer patients [7, 8]. Sultana et al. [7] reviewed 35 prior studies in a systematic review and identified individual barriers of socioeconomic status disadvantages, digital inequities, low patient motivation, and systemic factors of limited healthcare access, inadequate provider engagement, and lack of financial support. The authors identified solutions including financial incentives, culturally tailored interventions, digital engagement strategies and public health interventions including expanded health coverage policies, population-tailored interventions, digital solutions, and healthcare workforce training. None of the studies focused on cancer patient populations. Coleman et al. [8] performed a scoping review of 40 studies in community healthcare settings. Barriers to cessation included low literacy, competing demands and priorities, insufficient time, poor access to cessation products, lack of access to cessation services, limited workforce, lack of motivation, and lack of cessation readiness. Likewise, none of these reviewed studies included populations with cancer.

Because barriers to cessation can vary considerably from patient to patient, we hypothesized that a personalized medicine approach, allowing individual patients to choose from a menu of cessation strategies to meet their own preferences and needs, might improve patient engagement and tobacco abstinence. Based on this hypothesis, we developed a menu of diverse tobacco cessation services that addressed educational deficits and provided motivational aids, individual and group support, and behavioral interventions. This menu of services we called the personal pathway to success (PPS) program. We implemented this PPS program in patients scheduled to have surgery at City of Hope (COH). This communication summarizes the results of the PPS program in patients in whom we now have 12-month follow-up data on tobacco use.

## Methods

### Hypothesis

The hypothesis was that a personalized patient-centric approach, allowing an individual patient to choose from a menu of cessation strategies to meet their own needs and interventions acceptable to him/her, improves patient tobacco cessation.

### Aims

#### Primary aim

To develop the PPS program and compare cessation outcomes among cancer patients who participate versus patients who do not participate.

#### Secondary aims

To compare cessation outcomes between patients who are from minority populations versus those who are not from minority populations, by gender, and by age.

To evaluate the use of evidence-based or specialist-selected supplemental tobacco cessation services by cancer patients.

### Patient eligibility and intake

Each patient, when initially seen at COH, had tobacco use status (TUS) recorded in the electronic medical record (EMR, Epic). TUS was performed by medical assistants or nurses. The TUS included patient-reported use of cigarettes, cigars, or vaping, number of cigarettes used, day tobacco last used, and daily or intermittent tobacco use in the last 30 days. Current tobacco users were eligible to participate in the PPS program. Because outcomes of surgery are worse in patients who are current smokers [9], we initially implemented the PPS program at the surgery center at the COH Duarte campus. Current smokers who spoke English, or who spoke Spanish or Chinese (and who had appropriate interpreters), were offered the PPS program on the initial visit to the pre-anesthesia testing clinic (PATC) by tobacco treatment specialists (authors BG, AS, SY).

After the initial visit in the PATC, patients either agreed to participate in the full PPS (PPS participants) or declined participation or could not be contacted after the initial visit (PPS non-participants). The TTS attempted to contact non-participants up to 3 times to determine if they wanted to participate. If patients still wanted to be non-participants, or did not respond to the TTS phone calls, letters were sent to their primary care physicians urging them to advise cessation and prescribe NRT and/or varenicline or bupropion. All patients subsequently completed PATC pre-surgical testing and later received surgery.

### PPS core program

Prior to the PPS program, the standard approach to tobacco cessation was a referral by the treating physician of a patient who used tobacco, to the cessation program where a tobacco treatment specialist (TTS) would contact the patient and if the patient agreed, an appointment was made for cessation counseling with a TTS-certified nurse practitioner or pulmonologist who performed cessation counseling and prescribed cessation medications. The prior intake took 10 to 15 min.

The PPS program was implemented in September 2021 and expanded the initial contact with the patient by adding a longer motivational interview by the TTS with each patient. The motivational interview took 25 to 45 min and included assessment of the patient perception of smoking harm related to the current diagnosis, discussion of patient smoking behaviors, recognition of patient routines and smoking as a coping mechanism in the absence of healthy behaviors, patient understanding why change is needed and is beneficial for long-term health, and introduction of the PPS menu of supplemental tobacco cessation services.

To educate patients, we developed a tobacco cessation instructional video that was sent by email to each patient. All patients (unless they could not be reached by phone and did not have an email address or declined to be sent the video) received the cessation video. The instructional video included the dangers of tobacco or nicotine use, information on treatments, importance of cessation, benefits of quitting, the 6-step guide for how to quit, importance of professional assistance, and contact information for the tobacco cessation program.

During the preparative phase, we evaluated the instructional video in an initial group of patients who completed a survey to evaluate their knowledge and self-assurance on a 5-point Likert scale. After viewing the video in 13 patients, 11 (85%) said they were confident to be tobacco-free, with a mean score of 4.3 on a 5-point Likert scale. Of those 13 patients, 10 (77%) reported that they had gained the knowledge to be tobacco-free, with a mean score of 4.4 on a 5-point Likert scale.

Patients who agreed to participate in the core PPS program received a 60-min cessation counseling session delivered by a TTS-certified nurse practitioner (author JM) or a pulmonologist (author AS) in person or by telemedicine. Nicotine replacement therapy (NRT) and/or varenicline or bupropion were prescribed by physicians or nurse practitioners to all agreeable patients. NRT included nicotine patch and/or nicotine gum. Patients who could not afford pharmacotherapy were referred to our charity program which then provided medications.

### PPS supplemental tobacco cessation services

In addition to the core program components, patients who agreed to participate in the full PPS program were offered the supplemental PPS services. Supplemental cessation services were selected based on guidelines (e.g., National Comprehensive Cancer Network [NCCN] guidelines [10]), evidence-based interventions, or consensus among the tobacco treatment staff. These services included buddy or family support [11], KickItCA [12], Smoke Free [13], build a quit plan [13], Veterans Smoke Free [14], sign a commitment contract [15], relaxation and guided imagery [16], self-hypnosis [17], stress management APPs [18], spiritual care [19], Center for Disease Control (CDC) e-cigarette fact sheet [20], tobacco unfiltered podcast [21], use of cinnamon stick [22], use of bubble blowing [23], use of 5Ds [24], and use of 7 self-help strategies [25].

PPS participants worked with a TTS to determine which of the supplemental cessation services in the PPS program the patient was willing to use. Participation in the support groups was not restricted to City of Hope (COH) patients. Friends, family members, and also other patients from non-COH cessation programs were all welcomed to the support groups which were conducted in English, Spanish, and Chinese. Since social or familial gatherings can be a trigger to tobacco use in patients, the support group experience was a method to give instruction to friends and families to abstain from tobacco use while in the presence of the patient, and instead to encourage patient abstinence or tobacco reduction.

### Program evaluation

Participating patients were evaluated for use of PPS supplemental cessation services and for tobacco use (using the TUS) prior to surgery, at 30 days post-surgery, and at 3, 6, 9, and 12 months after surgery. Non-participants were only evaluated with a TUS before surgery and at 12 months post-surgery. Use of PPS supplemental services was evaluated by TTS interviews with patients. Patients self-reported their use of tobacco products. Data were collected from the electronic medical record (EPIC) and patient interviews by a TTS using an Excel worksheet. Data were stored in the Department of Population Science by the TTS in a secure computer backed up on Share Point and further stored on One Drive cloud storage. Analyses were performed by the authors.

### Program definitions

Total abstinence was defined as no use of tobacco and no vaping for at least 7 days. Abstinence from combustible tobacco use was defined as no cigarette use for at least 7 days, or no cigarette use but with use of vaping only. Reduction in number of cigarettes used, even without cigarette abstinence, was also evaluated. We defined total benefit as abstinence, abstinence from cigarette use but with continued vaping, and/or reduction in the amount of cigarette use. Reach of the PPS program was defined as the number of patients who completed a TUS, had motivational interviewing and/or were sent the tobacco cessation video.

### Statistics

Statistical comparisons were performed using chi-square or Fisher’s exact statistics (https://www.socscistatistics.com/tests/chisquare2). Statistical assistance was provided by Paul Frankel.

### IRB and consent

The PPS program was designed as a quality improvement project and was approved by the COH Investigational Review Board (IRB number 19201). Informed consent was not required by the IRB. Clinical trial number was not applicable.

## Results

### Patient characteristics

In this communication, we performed a detailed evaluation of the initial cohort of 70 patients accrued to the PPS program from September 2021 through September 2022 and in whom we had completed 12-month tobacco use survey (TUS) reporting (through November 2023), usage of individual PPS services, and program evaluations (Table 1). Insurance re-directed 7 of these patients (10%) for follow up by contracted providers not in the COH network. Of the remaining 63 evaluable patients, 35 (56%) agreed to participate in the full PPS program. Twenty-eight patients (44%) were non-participants. Of the non-participants, 27 patients did complete the tobacco use survey, undergo a motivational interview, and/or receive the tobacco cessation video (96.4% of the non-participants).

**Table 1:** Patient characteristics

	Total	Agreed to participate	Non-participant
Numbers of patients	70		
Insurance re-directed to non-COH provider	7 (10%)		
Evaluable patients contacted	63 (90% of all patients)	35 (56% of evaluable patients)	28 (44% of evaluable patients)
Age			
Median (years)	62	61	64
Range	29–78	29–78	38–75
Gender			
Female	31 (49%)	20 (57%)	11 (39%)
Male	31 (49%)	14 (40%)	17 (61%)
Non-conforming	1 (2%)	1 (3%)	
Race/ethnicity			
Black	5 (8%)	3 (9%)	2 (7%)
Asian	8 (13%)	5 (14%)	3 (11%)
Hispanic	10 (16%)	6(17%)	4 (14%)
Middle Eastern	8 (13%)	5 (14%)	3 (11%)
Native American	1 (2%)	0 (0%)	1 (4%)
Non-Hispanic white	29(46%)	14 (40%)	15 (54%)
Declined/unknown	2 (3%)	2 (6%)	0 (0%)
Total Minority	32 (51%)	19 (54%)	13 (46%)
Insurance			
PPO/EPO	12 (19%)	6 (17%)	6 (21%)
HMO	5 (8%)	1 (3%)	4 (14%)
ACA	7 (11%)	3 (9%)	4 (14%)
Medicare	32 (51%)	19 (54%)	13 (46%)
MediCal (Medicaid)	5 (8%)	5 (14%)	0 (0%)
Self-pay/unknown	2 (3%)	1 (3%)	1 (4%)

There were no differences between participants and non-participants with regard to gender (*p* = 0.20), being from a minority group (*p* = 0.31), or having no insurance or MediCal versus other insurance (*p* = 0.17)

Motivational interviews were completed in 56 of 63 evaluable patients (acceptability rate 92%). Only 1 patient could not be reached by phone after 3 attempts and did not have an email for sending the cessation video and educational information (4% of non-participants). The overall reach was 63 out of 63 evaluable patients (100%).

Among the 63 patients, gender was 49% female and 49% male. Although more females agreed to participate (57%) compared to males (40%), the difference was not statistically significant (*p* = 0.20). Median age was 61, similar in participants and non-participants.

Diagnoses in the 63 evaluable patients were carcinoma of the breast (9), colon (4), prostate (5), endometrium (4), stomach (4), kidney (2), brain mass (4), and other miscellaneous cancers or masses (19). Procedures which were performed were mastectomy (8), hysterectomy (5), colectomy (4), prostatectomy (4), pulmonary lobectomy or biopsy (4), gastrectomy (3), parotidectomy (2), or other procedures or biopsy (29).

Of the 63 evaluable patients, 32 (51%) were patients from a minority group. Although we expected to have fewer patients from a minority group who would agree to participate, acceptance of participation was at least as high in patients from a minority group, 20 out of 32 (63%) patients from a minority group, compared to 14 participants out of 29 (48%) who were non-Hispanic white patients (*p* = 0.26).

All but 2 patients were insured. This is likely due to insurance review of patients’ insurance status at City of Hope before scheduling surgery. Every patient with MediCal (Medicaid) insurance agreed to participate, but 4 out of 5 patients with health maintenance organization (HMO) insurance were non-participants. There was no difference in participation in patients with no insurance or MediCal insurance, versus patients with other insurance (*p* = 0.17).

### Effectiveness of the PPS program

Effectiveness of the PPS program participation was measured (Table 2). Abstinence among full PPS participants increased from 3% at initial contact by the TTS up to 17% 2 days pre-surgery (*p* = 0.11), 29% 30 days post-surgery (*p* = 0.006), 35% 3 months post-surgery, 35% 6 months post-surgery, 31% 9 months post-surgery, and 42% 12 months post-surgery (*p* = 0.0001). Among non-participants, abstinence also increased from 7% at initial contact up to 17% at 12 months post-surgery (*p* = 0.3).

**Table 2:** Effectiveness of the PPS program

Time of evaluation	Initial	Initial	2 days pre-surgery	30 days post-surgery	12 months post-surgery	12 months post-surgery
	PPS participant	Non-participant	participant	Participant	Participant	Non-participant
Number of patients evaluated	35 (100%)	28 (100%)	35 (100%)	35 (100%)	31 (89% of initial participants)	18 (64% of initial non-participants)
Tobacco use survey						
Abstinent	1 (3%)	2 (7%)	6 (17%)	10 (29%)	13 (42%)	3 (17%)
Vaping only	6 (17%)	1 (4%)	6 (17%)	5 (14%)	4 (13%)	1 (5%)
Smoking	28 (80%)	25 (89%)	23 (66%)	20 (57%)	14 (45%)	14 (78%)
Lost to follow-up	0	0	0	0	1 (3%)	7 (39%)
Deceased	0	0	0	0	3 (10%)	3 (17%)

Significance: In participants, compared to abstinence at initial contact, abstinence at 30 days *p* = 0.006; abstinence at 12 months *p* = 0.001

In participants compared to non-participants, abstinence at 12 months *p* = 0.11; abstinence from combustible tobacco use at 12 months *p* = 0.037

Comparing abstinence among participants versus non-participants, while there was a strong trend for higher 12-month abstinence of 42% among participants versus 17% among non-participants, this was not statistically significant (*p* = 0.11). The frequency of abstinence from combustible tobacco use (abstinence or only vaping) at 12 months was higher in participants 55%, compared to non-participants 22% (*p* = 0.037). Of the non-participants who achieved abstinence at 12 months, none received NRT or varenicline or bupropion, suggesting that the motivational interview plus cessation video alone may have had a beneficial effect on long-term patient tobacco cessation.

In evaluating abstinence at 12 months, there was no association with gender. Among females, 8/18 were abstinent, compared to males 5/12 (*p* = 1.0). Surprisingly, among patients 65 years old or younger, 12/19 were abstinent, compared to only 2/12 patients over 65 (*p* = 0.02). There was no association with race/ethnicity. Among non-Hispanic white patients abstinence was 9/19 compared to abstinence in patients from a minority group 4/10 (*p* = 1.0).

We evaluated the total benefit of the full PPS program (defined as abstinence, use of vaping only, or reduction in cigarette use) at 12 months post-surgery (Table 3). Among full PPS participants, 21 patients had benefit (68%), while 10 patients continued to have same or increased tobacco use. Among non-participants, 7 patients had benefit (39%), but 11 patients continued same or increased tobacco use. While there was a trend for increased total benefit among full PPS participants, this was not statistically significant (*p* = 0.07).

**Table 3:** Total benefit of PPS in reducing tobacco use

	Participants	Non-participants
Time of evaluation*	12 months	12 months
Number of patients	31	18
No combustible tobacco use (abstinent or vaping only)	17 (55%)	4 (22%)
Still smoking	14 (45%)	14 (78%)
Reduced cigarette usage	4 (13%)	3 (17%)
Mean reduction of usage	52%	48%
No change in usage	9 (29%)	10 (56%)
Increased usage	1 (3%)	2 (11%)
Total benefit (abstinent, vaping only, or reduced smoking)	21 (68%)	7 (39%)

*Time of evaluation measured from initial registration usage or pre-op day 2 usage, to 12-month evaluation

Significance: In full PPS participants compared to non-participants, total benefit *p* = 0.07

### Use of PPS supplemental services

The frequency of use of the PPS supplemental cessation services is shown in Table 4. At least 1 Essential Core Service was used by each PPS participating patient, and 30 of those patients (86%) received the nurse practitioner–delivered cessation consultation. Twenty-six participating patients (74%) received nicotine replacement treatment (NRT). Twenty-three participating patients (66%) also attended a COH group support meeting.

**Table 4:** Usage of PPS program components. Usage of each program component according to patient participation

	Patients who agreed to participate	Patients who were non-participants
Total number of patients	35	28
COMPONENT USED		
Motivational interview*	33 (94%)	25 (89%)
Tobacco cessation video*	33 (94%)	25 (89%)
ESSENTIAL CORE SERVICES		
Used at least 1 service	35 (100%)	27 (96%)
Consultation with cessation provider	30 (86%)	0 (0%)
Received NRT*	26 (74%)	11 (39%)
Received varenicline or bupropion (V/B)*	19 (54%)	4 (14%)
Received both NRT and V/B*	16 (46%)	2 (7%)
Received any NRT or V/B*	30 (86%)	12 (43%)
Had tobacco use survey	35 (100%)	28 (100%)
Attended COH group support	23 (66%)	0 (0%)
Received individual cessation support	27 (77%)	0 (0%)
SUPPORT SERVICES		
Used at least 1 service	32 (91%)	0 (0%)
Buddy support (friend or member from COH peer support group)	23 (66%)	0 (0%)
Angel support (family member)	21 (60%)	0 (0%)
Non-COH support	18 (51%)	0 (0%)
Phone support or phone APPs (Kick It CA)	17 (49%)	0 (0%)
Text support (SmokeFree, Kick It CA Text)	11 (31%)	0 (0%)
Online live chat (kickitca.org, cancer.gov)	10 (29%)	0 (0%)
Web resources (smokefree.gov, veterans.smokefree.gov)	17 (49%)	0 (0%)
COMMITMENT ADHERENCE TOOLS		
Used at least 1	16 (46%)	0 (0%)
Built a quit plan	16 (46%)	0 (0%)
Signed a written commitment contract	0 (0%)	0 (0%)
DAILY COPING STRATEGIES		
Used at least 1	27 (77%)	0 (0%)
Reviewed 5 D’s	27 (77%)	0 (0%)
Reviewed 7 self-care strategies	27 (77%)	0 (0%)
Instructions for cinnamon stick/bubble blowing	22 (63%)	0 (0%)
Reviewed COH relaxation/guided imagery video	22 (63%)	0 (0%)
Reviewed COH smoking cessation hypnosis video	16 (46%)	0 (0%)
Reviewed audiobook education (Libby)	15 (43%)	0 (0%)
Reviewed stress management APPs**	15 (43%)	0 (0%)
Discussed spiritual care	15 (43%)	0 (0%)
OTHER EDUCATIONAL TOOLS		
Used at least 1	5 (14%)	0 (0%)
Non-COH educational handouts	5 (14%)	0 (0%)
Reviewed E-cigarette CDC fact sheet	0 (0%)	0 (0%)
Reviewed modular videos	0 (0%)	0 (0%)
Watched tobacco unfiltered podcast	0	0 (0%)
OTHER NON-COH SERVICES USED	14 (40%)	0 (0%)

*Use of these services was evaluated at baseline. Use of all other services was evaluated over the 12 months after baseline

Medications other than NRT, varenicline or buproprion, were not evaluated

**APPs, e.g., Headspace, InsightTimer

The TTS supported each non-participant by sending a letter to the attending physician urging NRT and varenicline or bupropion, as well as asking the physician to urge tobacco cessation with the patient. Among non-participants, 11 patients (39%) received NRT and 4 patients (14%) received varenicline or bupropion.

Among participants, 32 patients (91%) received at least 1 PPS Support service. Most frequently used were “buddy” support by friends or members of a COH peer support group (66%), “angel” support from a family member (60%), or phone support through phone apps such as KickItCA (49%). Web resources were also commonly used (29–49%) including smokefree.gov, text support, online live chat support, or veterans.smokefree.gov.

A personalized quit plan was built by 16 patients (46%). These patients used a cessation adherence tool. Surprisingly, no patient signed a written cessation commitment contract.

At least 1 Daily Coping Strategy was used by 27 participating patients (77%). Each of these patients reviewed the 5 D’s (distracting, delaying, drinking water, deep breathing, and discussion) and also the 7 self-care strategies (healthy coping, healthy eating, regular exercise, taking medicine, monitoring, reducing risk, and problem solving). Most patients but not all also received other services as in Table 4.

Fourteen patients (40%) used non-COH services. These services included distraction by reading books, other relaxation techniques, use of puzzles and games, dog-walking, crocheting or sewing, bicycle riding, taking time with grandchildren, talk therapy, piano music lessons, completing home or boat improvement projects, watching YouTube, exercising, walking, art projects, and measuring or logging financial savings. They also included non-COH support groups (e.g., Nicotine Anonymous, Celebrate Recovery) and other web resources (becomeanex.org, truthinitiative.org, undo.org, American Cancer Society, American Lung Association, CDC, or World Health Organization [WHO]).

Individual PPS supplemental services were evaluated for their association with 12-month abstinence rates. Although none was statistically significant, marginal associations were observed for use of a relaxation/stress reduction APP (*p* = 0.07), use of online chat (*p* = 0.11), and use of 5Ds or 7 self-care strategies (*p* = 0.19 each). All other associations showed no association (*p* > 0.25 each).

Overall, 417 individual PPS supplemental services were used by the 35 participating patients. The mean number of services received was 11.9 per patient. Among non-Hispanic white patients, a mean of 11.1 services per patient was received, while among patients from a minority group, a mean of 12.0 services per patient was received. Evaluation of use of services by race or ethnicity of patients showed mean use of services among Hispanic patients was 16.5, among Black patients was 10.0, among Asian patients was 13.7, and among Middle Eastern patients was 6.6. For the 28 patients who received at least 1 service, mean number of services received was 15.4 services per patient, 16.0 services per non-Hispanic white patient, and 15.2 services per patient from a minority group. Use of supplemental services was different according to gender. Females used more services (mean 15.0) compared to males (mean 7.8, *p* = 0.005).

We evaluated 12-month abstinence rates according to amount of use of PPS supplemental services. In participating patients who received at least 1 PPS supplemental service, abstinence at 12 months post-surgery was 12/25 (48%), compared to abstinence at 12 months post-surgery in 4/24 (17%) in participating or non-participating patients who received no PPS supplemental service (*p* = 0.03). If patients received more PPS supplemental services than the mean (11.9 services per patient), abstinence rate was 10/20 (50%), versus patients who received fewer services than the mean whose abstinence rate was 4/13 (31%). While this trend favored use of more services to stay abstinent at 12 months post-surgery, this was not statistically significant (*p* = 0.31).

## Discussion

National guidelines, such as CDC and NCCN guidelines, have provided tips and resources which clinicians and providers can choose to use with patients [10, 26]. In contrast, the PPS program allows the patients themselves to select which supplemental cessation services to use.

Our data suggest that allowing patients to choose their cessation services was associated with a high abstinence rate. Patients who used both core and PPS supplemental services had a numerically increased abstinence rate at 12 months (42%) compared to patients who did not use supplemental PPS services (abstinence rate 17%), but this did not reach statistical significance (*p* = 0.11). A larger number of patients are necessary. The use of motivational interviews, instructional videos, and cessation medications in 89% of non-participating patients may have decreased the difference between participants and non-participants and accounted for the 17% abstinence rate at 12 months in non-participants. This is supported by the observation that among non-participants who were abstinent at 12 months, none received NRT, varenicline or buproprion.

Participating patients had a rate of abstinence from combustible tobacco use (abstinence from smoking) of 55% compared to non-participants whose rate was 22% (*p* = 0.037). There was a trend for greater use of PPS supplemental services to be associated with increased abstinence.

Barriers to cessation are common among populations and healthcare systems [27]. Others have identified the less-than-optimal reach and effectiveness in patients from minority populations, especially Black patients [28]. Our data showed that using a PPS approach, abstinence was at least as good in patients from minority groups as in non-Hispanic white patients.

Effectiveness of the PPS program compares favorably with other models of smoking cessation. M D Anderson clinicians reported 43.7% abstinence at 9 months in cancer patients [29] using medical consultation, 6–8 telephone counseling sessions and pharmacotherapy. The C3i multi-institutional study showed 18.4% effectiveness at 6 months among 28 comprehensive cancer centers [30]. A meta-analysis of 23 randomized controlled trials showed that a combination of pharmacological and behavioral interventions increased efficacy by 36% [31], and many of these interventions are included in the PPS program.

Sustained smoking cessation counseling and pharmacotherapy achieved a 34.5% quit rate at 6 months at Massachusetts General Hospital, Dana Farber/Harvard Cancer Center, and Memorial Sloan Kettering Cancer Center [32]. In peri-operative patients, smoking cessation was effective with 28.6% abstinence at 30 days after surgery [33].

Other models with enhanced services have been tested with variable results in randomized controlled trials. While some trials have not documented improved outcomes [34–36], other trials have shown benefit [37, 38]. Our results appear to be favorable and suggest that a randomized trial is warranted.

Tobacco cessation services are expensive and resource intensive [39] with a cost of $1989 per patient and an incremental cost to quit of $3906. Finding less resource-intensive methods to deliver more effective cessation to patients is an unmet need. The PPS program uses a TTS in conjunction with web-based resources and video education and cessation-promotion to accomplish objectives. Many of these PPS supplemental cessation services are not resource intensive. The PPS program might allow practices or institutions which are resource challenged to increase abstinence rates in patients.

Importantly, among patients from minority groups who were participants, abstinence at 12 months was achieved in 9/20 (45%). In this difficult to reach population of patients from minority groups, the PPS program achieved a high long-term abstinence rate. We will test this observation in additional settings and with larger numbers of patients.

Because of prior reports showing that relapses are frequent over time [40], tobacco cessation interventions must be studied over longer periods. Our evaluation of patients at 12 months helped identify patients at continuing need for cessation intervention. The PPS program allows patients to add more supplemental PPS services over time.

There are other options for implementation of cessation and individual workflow processes emphasizing quitlines and/or telephone counseling [41–43]. Mobile technology and machine learning may improve smoking cessation in the future [44].

Results from the ELEVATE innovative program demonstrated good results when using scripted interventions that rely on medical assistants and clinician actions [45]. This low-resource model offers advantages in simple implementation and sustainability but may have limitations in some individual patients. The PPS program offers the opportunity to individualize patient treatments and promote patient and family engagement.

## Limitations

Limitations of our study include the small sample size of 70 patients. This observational study was conducted in only one site, an academic center, and in only one venue, the PATC preoperative visit. Our evaluations of tobacco use were based on patient self-reporting, and we did not perform laboratory studies of nicotine or cotinine concentrations. This study was non-randomized. A randomized trial of the PPS program versus other methods of tobacco cessation would be necessary to prove that the PPS program is superior.

Since patients self-selected into the participant group or non-participant group, it is possible that participants were more likely to quit or had fewer prior quit attempts, which could lead to greater differences in outcomes. We emphasize that the non-participants also received motivational interviews and the instructional video, and many received cessation medications, which could mask differences between participants and non-participants. Measuring abstinence from combustible tobacco use (abstinence or only vaping) is not equivalent to complete tobacco abstinence, but it does represent harm reduction in evaluating total benefit of the program. There was a higher attrition rate in non-participants which could produce an imbalance in measured outcomes.

## Future directions

We intend to expand this into care clinics at the academic center and into affiliated community oncology centers and in larger numbers of patients. Future research will combine additional elements of different models to attempt to achieve improved outcomes and reduced costs. A randomized clinical trial will be considered.

## Conclusions

The PPS program is feasible and offers a personalized approach to tobacco cessation which promotes patient engagement to become tobacco-free over time. Patients from minoritized groups responded well to this approach. The PPS program reduced smoking in participants. If confirmed in subsequent studies, the PPS program may be an additional model to improve tobacco control and cessation in patients with cancer.

## Data Availability

The datasets are not publicly available due to patient confidentiality but are available from the corresponding author upon reasonable request.
